# Induction and Processing of the Radiation-Induced Gamma-H2AX Signal and Its Link to the Underlying Pattern of DSB: A Combined Experimental and Modelling Study

**DOI:** 10.1371/journal.pone.0129416

**Published:** 2015-06-11

**Authors:** Francesco Tommasino, Thomas Friedrich, Burkhard Jakob, Barbara Meyer, Marco Durante, Michael Scholz

**Affiliations:** 1 GSI Helmholtzzentrum für Schwerionenforschung, Department of Biophysics, Darmstadt, Germany; 2 Department of Radiation Oncology, Washington University School of Medicine, St. Louis, Missouri, United States of America; 3 Technische Universität Darmstadt, Institut für Festkörperphysik, Darmstadt, Germany; National Taiwan University, TAIWAN

## Abstract

We present here an analysis of DSB induction and processing after irradiation with X-rays in an extended dose range based on the use of the γH2AX assay. The study was performed by quantitative flow cytometry measurements, since the use of foci counting would result in reasonable accuracy only in a limited dose range of a few Gy. The experimental data are complemented by a theoretical analysis based on the GLOBLE model. In fact, original aim of the study was to test GLOBLE predictions against new experimental data, in order to contribute to the validation of the model. Specifically, the γH2AX signal kinetics has been investigated up to 24 h after exposure to increasing photon doses between 2 and 500 Gy. The prolonged persistence of the signal at high doses strongly suggests dose dependence in DSB processing after low LET irradiation. Importantly, in the framework of our modelling analysis, this is related to a gradually increased fraction of DSB clustering at the micrometre scale. The parallel study of γH2AX dose response curves shows the onset of a pronounced saturation in two cell lines at a dose of about 20 Gy. This dose is much lower than expected according to model predictions based on the values usually adopted for the DSB induction yield (≈ 30 DSB/Gy) and for the γH2AX foci extension of approximately 2 Mbp around the DSB. We show and discuss how theoretical predictions and experimental findings can be in principle reconciled by combining an increased DSB induction yield with the assumption of a larger genomic extension for the single phosphorylated regions. As an alternative approach, we also considered in our model the possibility of a 3D spreading-mechanism of the H2AX phosphorylation around the induced DSB, and applied it to the analysis of both the aspects considered. Our results are found to be supportive for the basic assumptions on which GLOBLE is built. Apart from giving new insights into the H2AX phosphorylation process, experiments performed at high doses are of relevance in the context of radiation therapy, where hypo-fractionated schemes become increasingly popular.

## Introduction

The **γ**H2AX fluorescence assay became one of the methods of choice for the detection of radiation induced DNA Double Strand Breaks (DSB), after it was shown in 1998 by Rogakou and co-workers that the H2AX histones are locally phosphorylated on Serine 139 in presence of a DSB [1], giving rise to the so-called **γ**H2AX foci, which can be observed and counted with a fluorescence microscope. **γ**H2AX foci counting offers high sensitivity in the low dose range down to few mGy [2], which is of interest for radiation protection and medical applications [3–6]. At the same time, it ensures high accuracy only up to doses of few Gy [7,8]. Thus, the use of flow cytometry to measure **γ**H2AX fluorescence intensity is a candidate to allow the use of the assay in an extended dose range up to several tens of Gy [9].

Among the different types of radiation induced DNA lesions, a primary role in cell killing is attributed to DSB. Specifically, the number of residual DSB observed late after irradiation, rather than the number of initial induced lesions, has been found to give indications concerning cell inactivation probability [9–13]. For instance damage clustering at different levels, i.e. nanometre [14–17] or micrometre scale clustering [18–21], is often associated to a higher severity of the induced damage, and thus to a prolonged persistence and enhanced lethality. However, these concepts are debatable, and a complete picture for the description of the pathways leading from DSB to cell killing is not yet available. The majority of the studies pointing in this direction are usually performed in a dose range typically below 10 Gy. Despite of this, there is increasing interest in the radiation therapy community to apply hypo-fractionated regimes in clinical practice [22]. These consist in the delivery of high doses to the tumour (20–30 Gy) in few fractions or even in a single shot. From this point of view, new interest arises in the investigation of biological effects induced after exposure to high radiation doses.

A consistent description of DNA damage and processing in an extensive dose range is also needed for the implementation of radiobiological models which can be used in treatment planning. The Giant LOop Binary LEsion model (GLOBLE) is a radiobiological model able to predict biological effects resulting from photon irradiation [23]. The model allows predicting DSB induction patterns in the context of the higher-order chromatin organization. Specifically, the genome is considered to be organized in so-called Giant Loops, each one involving about 2 Mbp of chromatin [24,25]. Thus, according to the presence of one or multiple DSB inside such loop structures, two different classes of DSB are defined and associated to a different damage severity. The same concept of DSB classification into iDSB and cDSB is also implemented in the Local Effect Model (LEM IV [26,27]) that allows predicting the effects of high-LET radiation based on the known response of a cell or tissue to low-LET radiation. This model is used in the framework of treatment planning for ion beam therapy [28,29].

In the framework of the validation of our modelling approach, we recently presented a DSB kinetic rejoining model based on the GLOBLE, which was able to consistently reproduce the dose dependence of DSB rejoining as measured by means of filter elution techniques after photon irradiation in a dose range between 10 and 200 Gy [30]. This shows that the gradually slowed-down processing of DSB after increasing photon doses can be well described solely based on the concept of DSB clustering at the micrometre scale. Since that analysis was based on the consideration of a single data set, additional work would be needed in order to further support those results. We thus decided to complement that study with experimental data obtained with more modern techniques, as for instance the **γ**H2AX assay. However, since such studies would imply the use of high doses in order to be sensitive to such effects, some practical issues have to be considered.

In fact, while the physical methods of detection allow the quantification of DNA fragments after irradiation (which can be linked to the number of induced DSB), the spatio-temporal characteristics of the **γ**H2AX signal suggest that it should rather be considered as an indirect marker of DSB presence [2]. While DSB start to be rejoined directly after induction, the **γ**H2AX fluorescence signal needs about 30–60 min to reach a maximum in the fluorescence intensity, before decaying over time [31]. This is a consequence of the H2AX phosphorylation taking place in an extended chromatin region surrounding the DSB. Thus, while DSB can be considered as a point-like event, the same does not hold for the **γ**H2AX foci, being characterized by a finite spatial extension [1,31].

The implications of the finite **γ**H2AX foci size in the scoring of DSB have been considered in the past, especially concerning high LET radiation, in experimental [32,33] and modelling [34,35] studies. Several mechanisms potentially leading to a reduced number of foci compared to the actual induced DSB have been proposed, as for instance the merging of close-by foci and/or the presence of multiple DSB in the same focus. These mechanisms are expected to play a substantial role after high LET irradiation, but in principle their conclusions should be applicable to the description of foci patterns resulting from high doses of low LET radiation as well.

Additionally, mechanisms have been recently proposed to describe the possibility of a three-dimensional accumulation of repair factors around the DSB, which would be responsible for a further extension of the phosphorylated region. In particular, according to the work presented by Muñoz et al [36], this would be related to the activity of DNA-PK bound to unwound chromatin ends, resulting in the activation of a chromatin region comprised between 2 and 10 Mbp. At the same time, Savic proposes a model where the spreading of H2AX phosphorylation is a consequence of a phospho-ATM (pATM) gradient around the induced DSB [37]. Obviously, these aspects do not represent a critical limitation for the practical use of the method, but they can affect the understanding of the experimental data, thus potentially resulting in controversial interpretations.

Here we present new experimental data, obtained by means of flow cytometry measurements of the **γ**H2AX fluorescence signal intensity after X-rays irradiation in an extended dose range, and compare them with a modelling analysis inspired by the GLOBLE approach. The aim was to analyse the dose dependence in DNA repair after low LET irradiation, thus contributing to the validation of the basic assumptions on which the GLOBLE model is built. At the same time, the use of the **γ**H2AX assay at high doses, initially motivated by our interest in investigating DSB kinetics of rejoining, revealed to be an interesting aspect on itself for the study of basic model parameters. Thus, full dose response curves were measured with flow cytometry, complemented by parallel confocal microscopy imaging, and then compared to model predictions. The influence of the spatial extension of the phosphorylated regions and of the DSB induction yield is widely considered and discussed. The potential role which can be attributed to 3D phosphorylation mechanisms is also taken into account. These aspects are found of general relevance for the interpretation and the understanding of experimental data obtained by means of the **γ**H2AX fluorescence assay. The kinetics of the **γ**H2AX signal has been investigated up to 24 h after irradiation with increasing X-rays doses up to 500 Gy, and a GLOBLE-based kinetic model is proposed to describe the observed signal. The gradually slowed-down disappearing of the **γ**H2AX signal at high doses is well described by the model. Together with our previous studies, this is supportive for the relevance of damage clustering at the micrometre scale in higher-order chromatin structures, in order to understand the biological effects induced by ionizing radiation.

## Methods

### Cell culture and irradiation

Chinese Hamster Ovary cells (CHO-K1, American Type Culture Collection) were cultivated using Ham's F12 medium (Biochrom) supplemented with 10% Fetal Bovine Serum (FBS) and 1% Penycilin/Streptomycin; a solution of 0.05% trypsin/0.02% EDTA was employed for trypsinization. Human skin fibroblasts (AG01522D abbreviated as AGD, obtained from Coriell Institute for Medical Research) were grown in EMEM with EBSS medium (Lonza), where 15% FBS and 1% Glutamine were added. A solution of 0.05% trypsin/0.1% EDTA was used for trypsinization. The cells were grown in culture flasks (25 or 75 cm^2^) at 37°C in incubators, where the atmosphere was composed by 95% air and 5% CO_2_. Cells were kept in exponential growth by regular sub-culturing.

**Fig 1:**
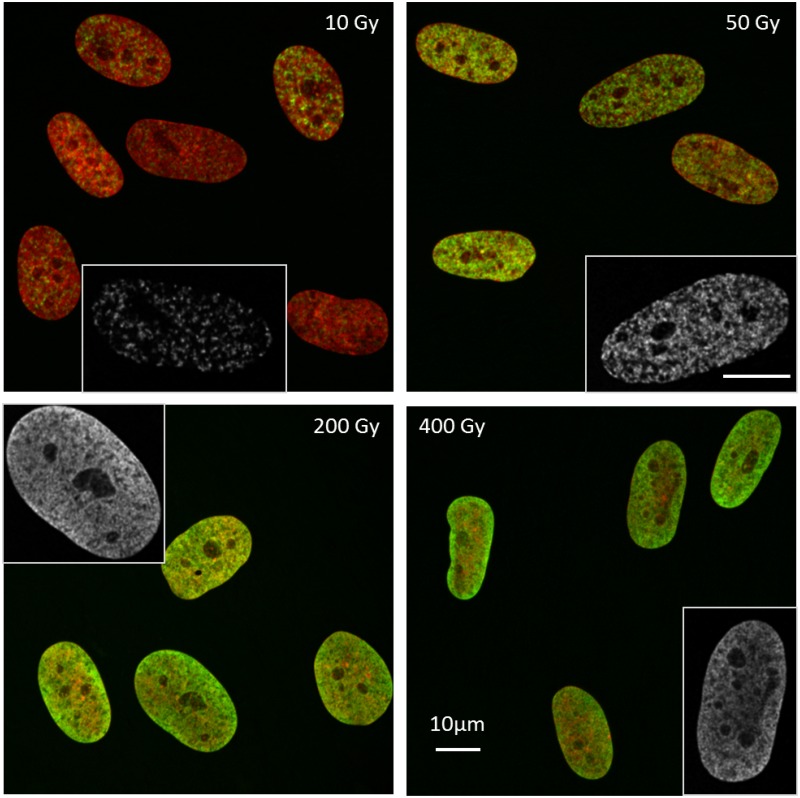
γH2AX fluorescence pattern at high photon doses. **γ**H2AX fluorescence pattern (green) of AGD cells as observed at the confocal microscope 1 h after high doses of X-rays irradiation. The “sum slices” Z-projection obtained with the ImageJ software is shown after deconvolution. DNA was counterstained with DAPI (displayed in red). The insert shows a single slice of **γ**H2AX in greyscale (deconvoluted) with a magnification factor of 1.5. Larger dark areas in the DNA staining which are free of **γ**H2AX fluorescence represent nucleoli [68].

**Fig 2:**
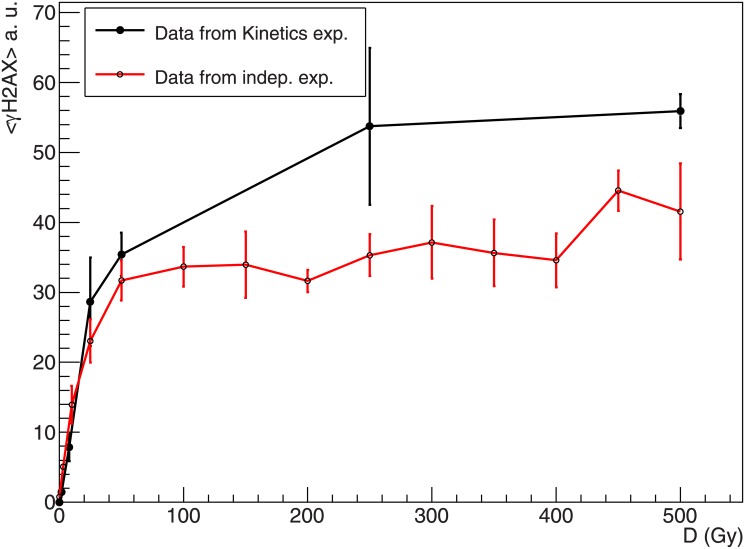
Measured γH2AX dose response curve up to 500 Gy. **γ**H2AX fluorescence intensity measured with flow cytometry 1 h after X-rays irradiation of AGD cells; the figure shows the dose response curve as extracted from the kinetics experiments (Fig 3) and the dose response curve resulting from independent measurements. Error bars show standard error of the mean for three or four independent samples. Fluorescence intensity is in arbitrary units.

**Fig 3:**
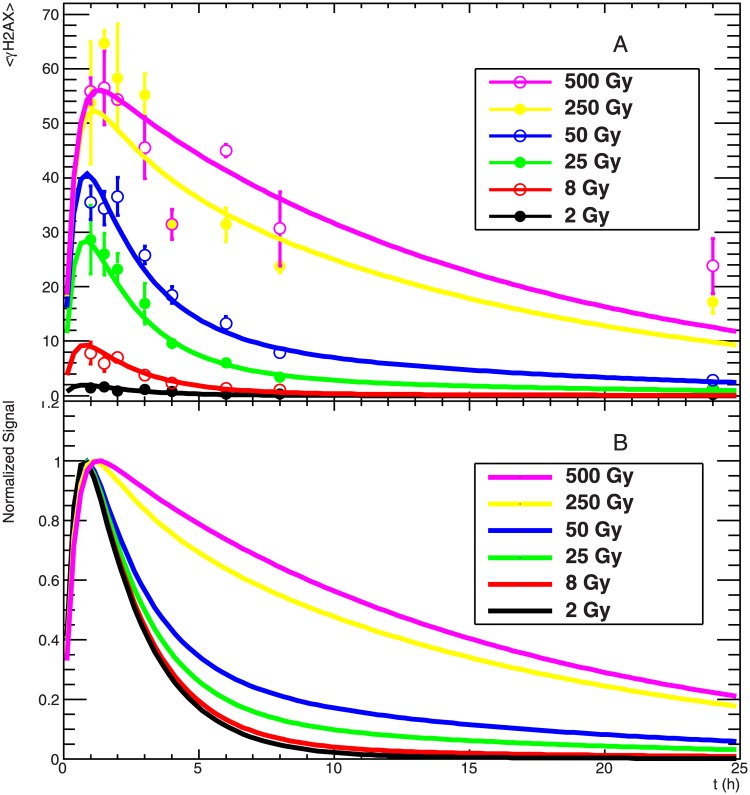
γH2AX signal kinetics in an extended dose range: experimental data and modelling analysis. Panel A: experimental data of flow cytometry measurements after irradiation with different X-rays doses (symbols) and corresponding application of the γH2AX kinetic model (lines); error bars show standard error of the mean for at least two independent samples. Lines show the result of the fit when input iDSB and cDSB are calculated according to Poisson distribution and assuming the standard domain size of 2 Mbp, considering also the 3D extension of the H2AX phosphorylation. Panel B: comparison between model predictions for the different doses investigated after normalization to their maximum value of the functions plotted in panel A.

Two different irradiation setups were employed. Flow cytometry experiments were conducted by using an x-ray tube working at 250 kV and 16 mA. A dose rate of 7 Gy/min was employed. Due to the need to reach high doses, a different x-ray tube working at 35 kV and 80 mA was adopted to perform experiments at high doses. This allows reaching a dose rate of about 100 Gy/min. Despite the different voltages, according to literature data no considerable difference is expected among the DNA damage patterns induced by the two x-ray qualities employed [38]. In order to ensure a homogeneous irradiation field, a single petri dish was placed at a distance of few cm from the tube exit window. With the aim to limit photon absorption, the medium level in the dish was reduced to 0.5 ml during irradiation. After irradiation, the samples were put in the incubators at 37°C. In both cases, dosimetry was performed with a calibrated ionization chamber. The dosimetry was performed at different distances from the source and at different currents, aimed at avoiding saturation effects of the ionization chamber. In order to test the homogeneity of the irradiation field Gafchromic films were also employed. This confirmed that the dose is released homogeneously at the biological samples. This setup was adopted to perform microscopy experiments shown in Fig 1, as well as for the dose response curve in Fig 2 and for the study of the **γ**H2AX kinetics at high doses (> 8 Gy) shown in Fig 3.

### Immunofluorescence staining and flow cytometry measurements

Cells were trypsinized 1 h after irradiation, fixed with a solution of 2% Paraformaldehyde (PFA) in Phosphate Buffer Solution (PBS), and permeabilized with 1 ml of Triton X solution 0.5% in PBS. 1ml of 0.4% BSA in PBS was added as blocking agent, and cells were kept at 4°C for a maximum of 7 days. After washing, a double antibody staining was used to detect the presence of **γ**H2AX molecules. Cells were resuspended in 100 μl of primary antibody (1:500 in PBS; mouse monoclonal anti-phospho-Histone H2A.X Ser139 primary antibody clone JBW301; Millipore) and incubated for 1 h at room temperature. Therefore, the samples were washed, the cells were resuspended in 100 μl of secondary antibody solution (1:400 in PBS; Alexa 488 goat anti-mouse F(ab)_2_ conjugate, Invitrogen, Karlsruhe, Germany) and incubated in the dark for 45 min at room temperature. Finally, 1 ml of DAPI solution (1:1000 in PBS) was added to obtain DNA staining as well. The fluorescence intensity of the samples was then measured at the flow cytometer after an incubation of at least 15 min in the dark at room temperature. In order to allow more accurate comparisons, samples belonging to the same experiments were stained and measured on the same day.

A PAS III cytometer produced by Partec was used in this study. The analysis of flow cytometry data was performed with the FloMax software, delivered together with the instrument. For each sample, the **γ**H2AX mean fluorescence intensity of G1 cells was quantified. The analysis has been restricted to G1 cells in order to minimize the bias introduced by spontaneous foci which can be measured in the S and G2 phases [39]. The background signal was quantified in unirradiated samples and then subtracted from the fluorescence levels measured in irradiated cells. An accurate quality control procedure was set-up, based on the use of polystyrene beads (3 μm Calibration Beads nr. 05–4008 and DNA Control UV Beads nr. 05–4020 from Partec) in order to check the stability of the instrument.

### Immunofluorescence staining and confocal microscopy

For the immunocytochemical staining experiments, cells were fixed in 2% PFA and permeabilized as described previously [32]. The same primary (dilution 1:500) and secondary (dilution 1:200) antibodies adopted for flow cytometry were employed. Counterstaining was performed with 1 μg/ml DAPI (displayed in red). Microscopic imaging was done on a Leica SPE confocal system using an ACS APO 63x 1.3NA oil immersion lens. Optical sections were recorded in increments of about 170 nm across the thickness of cells. The zoom factor was adjusted to yield pixels corresponding to 85 nm x 85 nm in lateral dimensions. Image analysis was performed with the ImageJ software.

### Modelling basics: the GLOBLE approach

**Fig 4:**
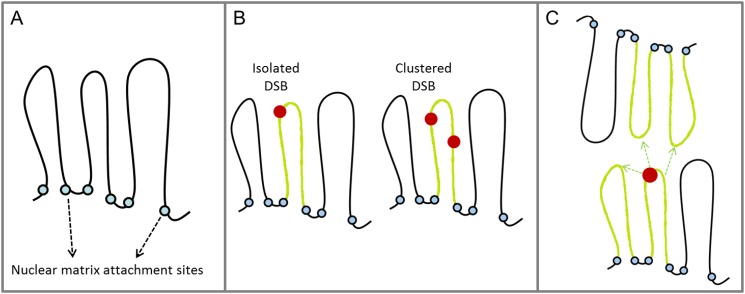
Modelling the H2AX phosphorylation in the context of Mbp-level higher-order chromatin structures. Starting from the basic assumption that chromatin loops involving about 2 Mbp of genome correspond to nuclear subunits that we define as *domains* (A), we make the hypothesis that a domain where at least one DSB is scored can be related to a phosphorylated loop, shown in green (B); importantly, no difference is expected in terms of fluorescence intensity depending on whether an iDSB or cDSB is induced, since in both cases we assume the whole loop being phosphorylated. In a further step, we consider the possibility that H2AX phosphorylation can randomly spread in a 3D fashion around the induced DSB. According to this approach, some of the domains surrounding the hit ones will produce γH2AX signal even without direct induction of DSB. This concept is schematically represented in panel C, where arrows indicate spreading of H2AX to additional domains surrounding the hit one.

The modelling work presented in this paper is based on the approach adopted in the GLOBLE for the description of radiation induced DNA damage [23]. The target is identified with the cell nucleus where the genome is stored. The nucleus is described as a cylinder having a radius of 5 μm and a total volume of 500 μm^3^. The higher-order chromatin organization is taken into account by considering the presence of Giant-Loops, also called domains here, each one involving about 2 Mbp of DNA [24,25] (see Fig 4A). Assuming the typical mammalian DNA content of about 6000 Mbp per cell and the cited size parameters, we come up with about 3000 domains of approximately 540 nm size length resembling the nuclear organization in the simulations. No difference is made between eu- and heterochromatin compartments of the nucleus. From now on, the words *loop* and *domain* will be used with the same meaning.

Based on the chromatin organization into loop structures, two classes of DSB are defined, namely isolated (iDSB) and clustered (cDSB) DSB according to whether only one or more than one DSB are simultaneously present in the same domain, respectively. Since the cDSB produce the loss of integrity of the loop structure, and they may even result in the loss of DNA fragments, they are considered to be more severe lesions compared to the iDSB.

### Modelling the H2AX phosphorylation: from DSB to hit domains

In our model we define as *hit domain* each domain where at least one DSB is scored. Then, we make the hypothesis that each hit domain can be associated to a loop where H2AX phosphorylation takes place following DSB induction. This is supported by literature data, suggesting a link between chromatin loops and H2AX histone phosphorylation [1,40]. We assume here that the phosphorylation process takes always place in the whole loop extension, independent on the presence of one or more DSB in the same loop. This means that no difference is made between iDSB and cDSB concerning their contribution to the fluorescence signal (see also Fig 4B).

In order to predict the numbers of induced iDSB and cDSB after photon irradiation, the Poisson distribution is adopted [23,27]. The average number of expected DSB per domain as function of the dose *D* can be defined as follows:

$$\lambda (D)=\frac{\alpha_{DSB}D}{N_{L}}$$ (1)

where *N*_*L*_ indicates the total number of domains and α_DSB_ represents the DSB induction yield. In line with the standard parameters adopted in the GLOBLE, *N*_*L*_ is chosen equal to 3000 while α_DSB_ is initially set to a value of 30 DSB per Gy and cell nucleus [8,41]. Importantly, in our approach the DSB induction yield includes implicitly all the lesions which are finally observed as a DSB. This means that prompt DSB as well as DSB originating from clustered non-DSB lesions are included into this parameter [42–44]. The average expected number of domains affected by iDSB (*n*_*i*_) and by cDSB (*n*_*c*_) at a given dose is then calculated with the following equations:

$$n_{i}(D)=N_{L}\lambda (D)e^{-\lambda (D)}\;n_{c}(D)=N_{L}(1-e^{-\lambda (D)}-\lambda (D)e^{-\lambda (D)})$$ (2)

Finally, the total number of hit domains for a given dose *n*_*T*_ results from the sum of the corresponding *n*_*i*_ and *n*_*c*_:

$$n_{T}(D)=n_{i}(D)+n_{c}(D)$$ (3)

With this approach, we can simulate the expected dose response curve after photon irradiation in terms of hit domains. In first approximation, this simulated curve should be comparable with the one measured with flow cytometry by means of **γ**H2AX fluorescence intensity. We use a normalization factor *x*_*fl*_ representing the average fluorescence per hit domain, so that the fluorescence signal is given by:

$$\gamma H2AX(D)=x_{fl}\cdot n_{T}(D)$$ (4)

### Modelling the possibility of 3D spreading of the H2AX phosphorylation

A further step is introduced in the model in order to take into account the possibility that H2AX phosphorylation can extend to domains surrounding the hit ones and where no lesion is induced, which we define here as *activated domains* (see Fig 4C). We assume that starting from a hit domain, the phosphorylation of H2AX can spread to some of the surrounding loops up to the point where a maximum of 10 Mbp of genome is activated [31,36]. As explained below, this is taken into account with a probabilistic approach. No principle difference is made between iDSB and cDSB concerning the spreading mechanism. From the practical point of view, having defined domains of about 2 Mbp size, this means that the phosphorylation extension can affect up to 4 domains surrounding the hit one. In the defined geometry each cubic domain has 26 neighbouring voxels (excluding domains belonging to boundary regions). For any given dose, the number of initial induced iDSB and cDSB is calculated, and a corresponding number of domain inside the nucleus is filled, in order to simulate the phosphorylation. Then the simulation algorithm proceeds through the following steps:

- The content of each domain belonging to the volume is checked;
- If the domain has no DSB, the algorithm checks the following voxel;
- If the domain has an iDSB or a cDSB, an integer random number *n*_*ext*_ between 0 and 4 is drawn to decide how many neighbouring domains are phosphorylated (in other words, the final genomic extension of **γ**H2AX foci is decided at this stage);
- *n*_*ext*_ of the 26 surrounding voxels are randomly activated to simulate the 3D extension of the phosphorylation starting from the hit domain;
- After the two random processes have been completed, the algorithm examines the following voxel, until all the voxels have been considered.

In order to increase calculation accuracy, the random process was repeated 500 times for each considered dose, and an average quantity was then computed. At this stage, we believe that the assumption of a uniform probability distribution defining the final extension of the phosphorylated region represents a reasonable approach for a first step approximation since no detailed experimental evidence is available. The same applies to the selection of which neighbouring domains are phosphorylated, assuming that there is no preferential direction for the spatial spreading. At the same time, we are aware of the fact that the choice of cubic domains is a simplistic approach. However, we think that in first instance this is a reasonable assumption to test the soundness of the model. Moreover, it is the genomic content of the domain rather than its exact geometry which is the most relevant aspect in GLOBLE. Therefore we do not exclude that more realistic geometry could improve the model, but we are confident that this would represent a second order effect which might be analysed in further studies. Starting from an initial average number of hit domains, the final outcome of the calculations is thus the average expected number of phosphorylated domains, including both directly hit or randomly activated domains.

### Modelling of the γH2AX signal kinetics

Aiming at the description of the **γ**H2AX signal kinetics, the approach recently presented in Tommasino et al [21] for the analysis of the DSB rejoining kinetics is taken as starting point and is further developed, in order to take into account the fact that H2AX phopshorylation is not an instantaneous process. Here the hypothesis is made, that H2AX phosphorylation affects the whole hit domain and takes place with the same kinetics independent on whether associated to an iDSB or to a cDSB, and can thus be parameterized with a common time constant. In contrast to other studies based on a bio-chemical approach [45,46], a detailed consideration of the phosphorylation mechanisms is beyond the scope of this work, therefore the role of kinases involved in the H2AX phosphorylation after irradiation (e.g. DNA-PK, ATM) is not explicitly considered. Importantly, the disappearing of the **γ**H2AX signal over time is not strictly related to the DSB rejoining but more generally reflects ongoing repair processes [31]. The mathematical expression used for the description of the **γ**H2AX signal kinetics is thus the following:

$$\begin{array}{l} \gamma \text{H2AX(t)}=n_{i}\cdot x_{fl}\cdot \frac{\tau_{\text{fast}}}{\tau -\tau_{\text{fast}}}\cdot {\text{(e}}^{-\frac{\text{t}}{\tau }\text{ln(2)}}-e^{-\frac{\text{t}}{\tau_{\text{fast}}}\text{ln(2)}}\text{)}+\\ +n_{c}\cdot x_{fl}\cdot \frac{\tau_{\text{slow}}}{\tau -\tau_{\text{slow}}}\cdot {\text{(e}}^{-\frac{\text{t}}{\tau }\text{ln(2)}}-e^{-\frac{\text{t}}{\tau_{\text{slow}}}\text{ln(2)}}\text{)} \end{array}$$ (5)

where *n*_*i*_ and *n*_*c*_ are calculated with the GLOBLE, and τ_fast_ and τ_slow_ refer to the half-lives of the fast and slow components of damage processing respectively. In addition, we need to introduce one more parameter in order to establish quantitative comparisons between the predicted frequencies of iDSB and cDSB and the measured signal. For this reason, the effective fluorescence per hit domain is considered by the term *x*_*fl*_. When the expression is used to fit experimental data as measured after irradiation with different doses or different radiation qualities, the three half-lives are considered as global fit parameters. The effective fluorescence is instead used as single-curve fit parameter. The frequencies of induced iDSB and cDSB represent input parameters of the model and their relative fractions define the complexity of the induced damage pattern, thus determining a faster or slower disappearing of the signal. The fit is based on a χ^2^ minimization on a linear scale, and error bars are considered. This model will be applied to the description of the **γ**H2AX kinetics after irradiation with different doses of X-rays.

## Results

### Flow cytometry measurements of γH2AX dose response curves

**Fig 5:**
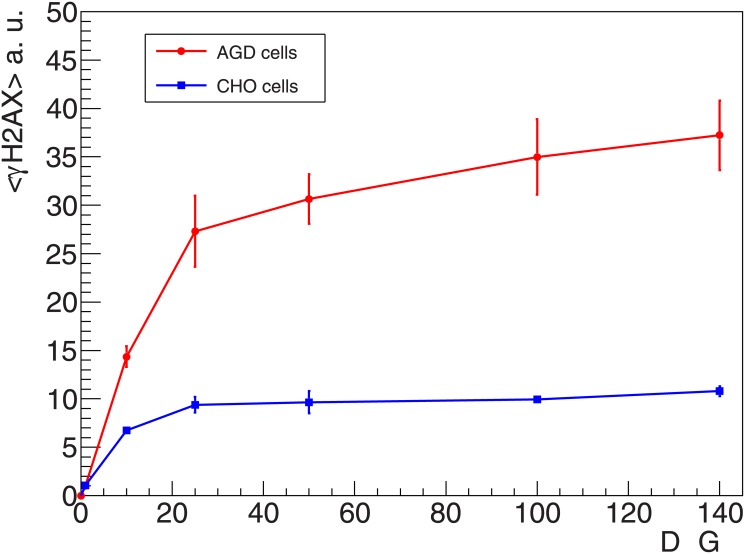
Measured γH2AX dose response curve. **γ**H2AX fluorescence intensity measured with flow cytometry 1 h after X-rays irradiation of AGD and CHO cells; error bars show standard error of the mean for four independent samples. Fluorescence intensity is in arbitrary units.

The dose response curve measured 1 h after irradiation, up to a dose of 140 Gy is shown in Fig 5 for AGD and CHO cells. Differences in the absolute values for a given dose between the two cell lines can be due to slight differences in genome content and/or in H2AX abundance, as well as in chromatin organization [1]. In both curves, a similar behaviour for increasing doses is observed. Specifically, the dose response starts with the trend of a linear rise in fluorescence intensity up to a dose of about 10–20 Gy, followed by gradual bending of the curves, expressing the transition into a “saturation region”. This bending seems to be more pronounced for CHO cells, and shallower for AGD cells. At the same time, in the high dose region the curve appears almost flat for CHO, while a low but continuous increase in fluorescence intensity is measured with AGD cells. Remarkably, according to results shown in the past and obtained by using physical methods for DSB detection (i.e. sedimentation, gel electrophoresis or filter elution techniques) no saturation would be expected in the induction of DNA lesions in the dose range of interest here [47,48].

In addition, in Fig 2 we show the results obtained with AGD cells by extending the dose response curve up to 500 Gy. The two curves refer to the dose response curve as extracted from the kinetics experiment (shown in Fig 3), as well as to the dose response curve resulting from independent measurements. Apart from some larger scattering observed at high doses, the values are in line with what observed in Fig 5. This essentially confirms that the observed saturation trend is maintained up to very high doses.

Dedicated tests have been performed, including variation of antibodies concentration and the use of antigen retrieval techniques, in order to exclude that the observed saturation results from technical artefacts. However, we observed no indications pointing at technical issues as explanation for the saturation behaviour.

### Microscopy analysis of the γH2AX fluorescence intensity at high doses

A parallel microscopy analysis was performed, aimed at the qualitative investigation of the **γ**H2AX distribution pattern in cells irradiated with high doses of X-rays. The resulting microscopy pictures are shown in Fig 1. The **γ**H2AX fluorescence patterns obtained after irradiation of confluent AGD cells with 10, 50, 200 and 400 Gy of X-rays are shown. We realize that while after 10 Gy it is still possible to observe a heterogeneous distribution of foci, although their high number hinders an accurate counting, after 50 Gy the **γ**H2AX seems to be distributed almost in the whole nucleus. However, the fluorescence is not homogeneous and we can distinguish spots of higher and lower intensity resulting in a more or less pronounced granularity of the staining, according to the specific cell under examination. A different fluorescence pattern is observed in the samples irradiated with very high doses, namely 200 and 400 Gy in this specific case, showing a much more homogeneous distribution of **γ**H2AX. This suggests that, in line with the low residual slope observed in the saturation region with flow cytometry, there is still room for further H2AX phosphorylation at such high doses.

These results are further supported by the direct comparison of flow cytometry data and integral fluorescence intensity as extracted from microscopy pictures which is shown in S1 Fig. This indicates that independent measurements performed with two techniques show the same tendency to saturation.

### Model predictions of hit domains

**Fig 6:**
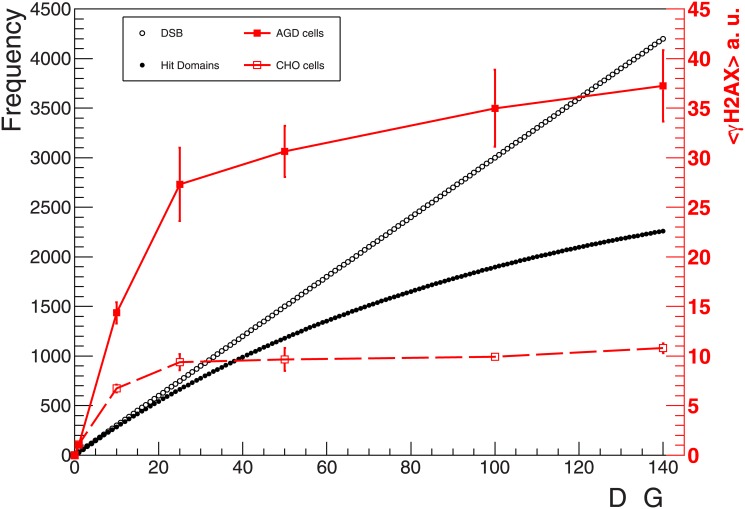
Modelling the γH2AX dose response: DSB vs hit domains. Comparison between expected numbers of induced DSB and hit domains as function of the dose; the calculations were performed assuming the standard input parameters of 30 DSB per Gy and cell nucleus and of 3000 domains (2 Mbp/domain).

The GLOBLE model was used to calculate the expected number of hit domains for a given photon dose (see Methods for details). Intuitively, given a fixed number of domains belonging to the nucleus, the onset of saturation is expected at some point in the dose response curve. This is a consequence of both clustering of DSB into the same domain, which in our approach is not expected to result in further phosphorylation, and of the number of not-hit domains decreasing for increasing doses. This is demonstrated in Fig 6, where simulated dose response curves of DSB induction and hit domains are compared. A DSB induction rate of 30 DSB/Gy and a foci size of about 2 Mbp were assumed in the calculations, in line with the basic and most common assumptions found in the literature [31,41,49]. We notice how the two curves progressively diverge for increasing doses. In particular, a tendency toward saturation is observed in the curve representing the number of hit domains as a function of dose.

When comparing the experimental curves shown in Figs 2 and 5 with the results plotted in Fig 6, we observe in both cases the general tendency toward saturation. At the same time, some discrepancies are evident. In particular, in the measured curves the saturation seems to be more pronounced and to arise at lower doses (about 20–40 Gy) as compared to the model predictions. Obviously, the actual value adopted for the DSB induction yield and for the domain size will influence the dose at which saturation is reached. A sensitivity analysis for these two parameters was performed and is shown in S2 Fig.

### Direct comparison of model predictions and experimental data

**Fig 7:**
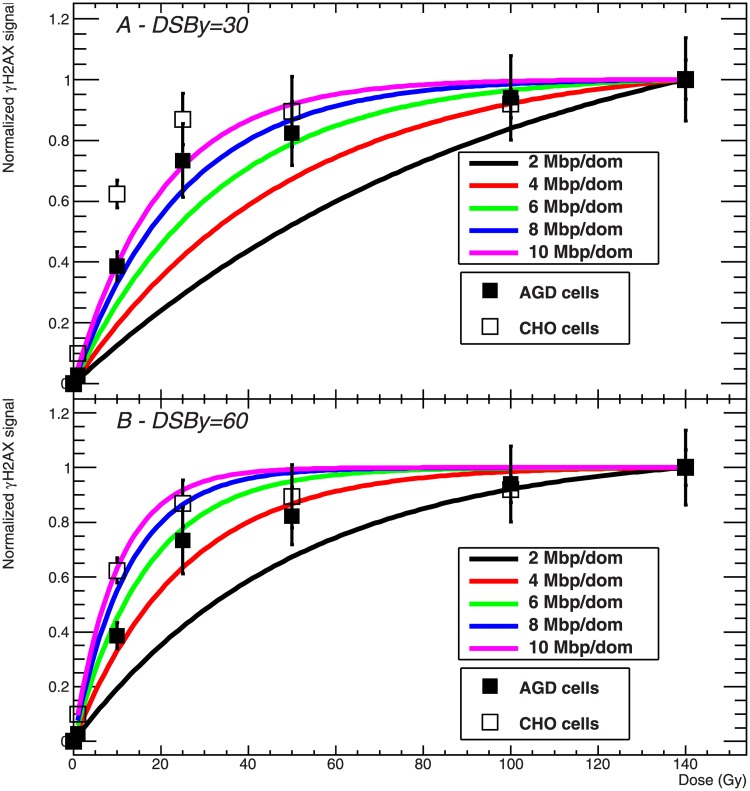
Comparison of measured γH2AX dose response curves to model predictions. Normalized **γ**H2AX dose response curve for AGD (full squares) and CHO (open squares) cells, and corresponding model predictions of hit domains for different domain sizes and a DSB induction yield of 30 DSB/Gy (A) and of 60 DSB/Gy (B).

**Fig 8:**
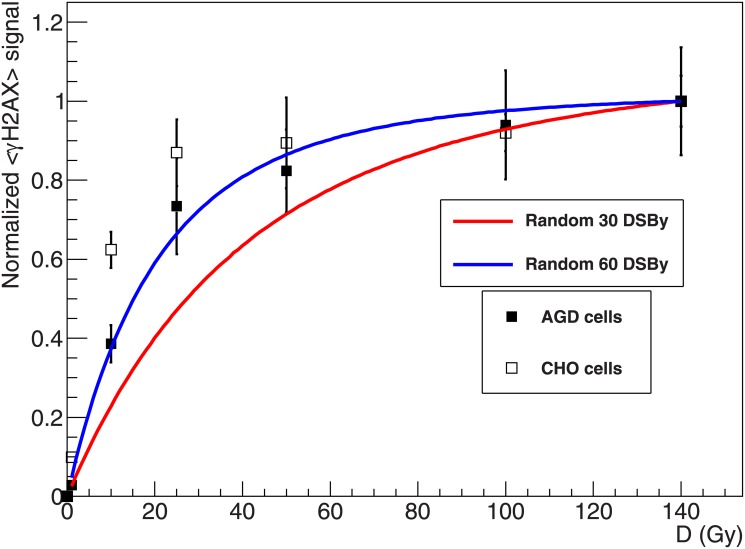
Comparison of measured γH2AX dose response curves to model predictions including the 3D spreading mechanism for H2AX phosphorylation. Normalized γH2AX dose response curve for AGD (full squares) and CHO (open squares) cells, and corresponding model predictions of phosphorylated domains assuming random extension of H2AX phosphorylation around the hit domain; a DSB induction rate of 30 and 60 DSB/Gy was considered.

In order to directly compare model predictions with the presented results, the experimental data were normalized by the fluorescence intensity measured at 140 Gy. These normalized experimental data (Figs 7 and 8) give information concerning the fraction of H2AX being phosphorylated at a given dose with respect to the value measured at 140 Gy. At this dose, CHO cells seem to have entered a comparably stable saturation level, while a residual slope is observed for AGD. This observation suggests that the H2AX content of CHO cells is activated with a higher effectiveness per induced DSB compared to AGD cells. In fact, having as reference the fluorescence intensity measured at 140 Gy, for CHO cells about 90% of this maximum level is already reached at a dose of 25 Gy, and this determines the flattened profile for higher doses, meaning that only a limited increase in signal intensity is expected when additional DSB are induced. At the same time, for AGD cells at a dose of 25 Gy the **γ**H2AX signal is at about 70% of the reference value, and therefore a higher slope is observed up to the maximum dose of 140 Gy.

The same approach is adopted for the analysis of model calculations of hit domains as function of the dose, by using the predicted values corresponding to 140 Gy as normalization factors. Thus, a qualitative comparison can be directly established between experimental and modelling results. Predicted curves are calculated for different combinations of model parameters. In Fig 7A the measured **γ**H2AX dose response curve for both CHO and AGD cells is shown together with model predictions calculated for different domain sizes and for the standard DSB induction rate of 30 DSB/Gy. We observe that the combination of 30 DSB/Gy and 3000 domains does not allow reproducing the experimental data. In fact, only a slight tendency to saturation is observed in the corresponding curve, which therefore diverges from the measured points. Concerning AGD cells, we see that a good agreement is obtained by assuming a larger domain size corresponding to a genome content of 8–10 Mbp/domain. In this case, the predicted curves nicely reproduce the initial rise of the signal up to about 25 Gy, and they also show the onset of a pronounced saturation as it is the case in the experimental data. When using domains of smaller size (4–6 Mbp), deviations from the measured points get smaller at high doses. On the contrary, in the case of CHO cells even the assumption of a large domain size is not enough to reproduce the initial steep increase of fluorescence which is measured in the experiments, although we can again observe a partial agreement for doses above 25 Gy if a DNA content comprised between 6 and 10 Mbp per domain is assumed.

In Fig 7B we tested model predictions for different domain sizes assuming also an increased DSB yield of 60 DSB/Gy. Noticeably, some improvements can be found in the description of both cell lines. In the case of AGD, there is no need to assume the presence of very large domains, since good agreement is already obtained with a genome content of about 4 Mbp/domain. Even more substantial are the changes for CHO cells, since we observe that the measured dose response curve is now comparably well reproduced if a domain size corresponding to 6–10 Mbp/loop is adopted.

From the comparison shown in Fig 7 we can finally conclude that reasonable agreement can be found between model predictions of hit domains and experimental measurements, although in general there is the need to assume a domain size and/or a DSB induction rate which deviate considerably from the canonical parameters suggested in the literature.

### Effects of 3D spreading mechanisms of H2AX phosphorylation

In Fig 8, the normalized experimental data are compared to model predictions of phosphorylated domains, considering also the 3D spreading mechanism. Shortly, we assume that some of the domains surrounding the hit can be phosphorylated even if no DSB is induced, and we call them *activated domains* (see Methods for details). Fig 8 shows that, even when the standard domain size is assumed, a pronounced bending toward saturation is now observed in the predicted curve. This tendency gets more pronounced if a higher DSB induction yield of 60 DSB/Gy is considered. Concerning AGD cells, the standard induction rate allows a reasonable agreement only at doses larger than 50 Gy, while the strong phosphorylation at low doses is not well reproduced. However, if the higher induction rate is assumed, model predictions fit very well to the experimental data. As before, deviations are in general larger for CHO cells. Although the model is not able to completely reproduce the measured curve, when an induction yield of 60 DSB/Gy is considered at least partial agreement is found concerning the initial increase up to about 5 Gy, and the high dose region comprised between 50 and 140 Gy where the predicted curve lies in the limits of experimental errors.

### γH2AX signal kinetics after irradiation with increasing doses of X-rays: experimental and modelling analysis

The kinetics of the **γ**H2AX fluorescence signal was investigated and then complemented by a modelling analysis. Here we focus on the consideration of the development of the fluorescence signal intensity over time after irradiation with increasing doses of X-rays comprised between 2 and 500 Gy. The measured data points (symbols) and the model results (lines) are reported in the upper panel of Fig 3A. A simultaneous fit was performed including all the experimental data, resulting in the determination of a phosphorylation half-life and of two decay half-lives for the processing of iDSB and cDSB. The parameter values are summarized in Table 1. The numbers of activated hit domains corresponding to induced iDSB and cDSB were calculated according to the algorithm where random expansion of H2AX phosphorylation is considered, which was shown to result in a better agreement to the measured dose response curves (Fig 8). Similar results compared to what is reported here were obtained by using only Poisson calculations of hit domains (see S2 Fig and S1 Table for fit parameters), in line with the expectation that the kinetics is defined by the relative fractions of induced iDSB and cDSB more than by their absolute values. These fractions are obviously dose-dependent but are not strongly affected by the specific algorithm used for the calculations. The main difference between the two approaches is in the absolute values of the *x*_*fl*_ parameters resulting from the fit. However, the global chi-square was only slightly higher when the simple Poisson statistics was employed.

**Table 1:** γH2AX signal kinetics: fit parameters for standard domain size (2 Mbp) and 3D spreading mechanism of H2AX phosphorylation.

D (Gy)	x_fluor_
2	0.0188 ± 0.0023
8	0.0245 ± 0.0017
25	0.0295 ± 0.0022
50	0.0271 ± 0.0028
250	0.0202 ± 0.0006
500	0.0200 ± 0.0003
τ	0.24 ± 0.04 h
τ_fast_	1.48 ± 0.10 h
τ_slow_	10.50 ± 0.59 h

Fit parameters resulting from the application of the **γ**H2AX kinetic model to the experimental data shown in Fig 3.

The fit results are in good agreement with the data, and produce half-life values in line with what is found in the literature [50,51]. Concerning the fluorescence per hit domain, it is remarkable to observe that, although used as single-curve parameters, we get similar values for the different doses considered. This is supportive for the hypothesis that this parameter should be dependent in first approximation only on the cell line but not on the dose.

In the figure we can observe that for increasing doses the maximum fluorescence intensity increases and is reached at a later time point, while the signal persists for a longer time. The comparison of the kinetics can be better appreciated when looking at Fig 3B. Here a plot is shown where the fit functions plotted in the upper panel are normalized to their maximum value. This normalization step allows a direct comparison of the signal kinetics and shows a gradually delayed disappearance for increasing doses, which in the context of our model is only due to a gradually enhanced proportion of induced cDSB. These results support the hypothesis that increasing photon doses results in a higher fraction of induced cDSB, corresponding to damage of higher severity compared to iDSB. This represents one of the basic concepts of the GLOBLE model, and in general is supportive for the biological relevance of micrometre-scale clustering of DSB.

## Discussion

Aiming at contributing at the experimental validation of GLOBLE, the study of the **γ**H2AX induction and processing in an extensive dose range was initially motivated by the interest in investigating DSB repair kinetics after increasing doses of photon radiation. In fact, this represents an important end point to analyse the relevance of micrometre scale damage clustering, which is a key aspect of GLOBLE. However, preliminary experiments showed the onset of a saturation tendency at doses around 20 Gy in the **γ**H2AX dose response curves as measured with flow cytometry (Figs 2 and 5). This was in contrast with what expected according to basic theoretical predictions (Figs 7 and 8), and thus triggered our interest in a better understanding of the H2AX phosphorylation process, as well as in the study of the impact of basic model parameters.

Our results could help to better elucidate the relation between induced DSB and **γ**H2AX foci, which despite the large use of the assay is not yet fully understood. At the same time, they are also supportive for the importance of damage clustering at the micrometre scale, thus contributing to the work carried out by our group for the validation of the GLOBLE basics. The relevant aspects of our analysis will be discussed in the next paragraphs.

### Dose dependence in DSB processing

Many attempts have been performed over the years, trying to identify the presence and the relevance of dose dependence in DSB processing after photon irradiation. For example, in one of the earliest investigations performed by Blöcher et al. using the sedimentation technique [47], Ehrlich Ascite tumour cells were irradiated with increasing doses of X-rays, and the initial dose response curve was compared with the one measured 24 h after irradiation. In the latter, a tendency toward a quadratic increase is observed. Similar results have been obtained also with the use of gel electrophoresis techniques by different groups [52,53]. Of particular interest in this context is the data set presented by Cucinotta et al., where the dose dependence of V79 cells is investigated by measuring with filter elution techniques the rejoining up to 3 hours after irradiation [54]. In this case, γ-rays were employed to deliver doses between 10 and 200 Gy, and a slowing-down is observed in the rejoining curves for increasing doses. As mentioned above, we were able to successfully describe these data with our DSB kinetic rejoining model [30].

In the more recent study by Neumaier et al. [55], the effects on the repair kinetics of increasing photon doses between 0.1 and 2 Gy were investigated. Despite the low doses, a tendency to dose dependence was observed in the kinetics of foci disappearing. This observation was associated to local dose effects which could take place when using the same radiation quality. One possibility would be that multiple DSB are simultaneously present in the same repair centre. The clustering of DSB at the micrometre scale within domains has been recently also exploited by Vadhavkar et al [35]. Based on this concept, they could interpret the induction of foci along high energy charged particle tracks.

For the sake of completeness, we also have to mention that there are at least two published studies where physical methods (i.e. gel electrophoresis elution) were employed to perform the experiments and where no clear dependence on the dose was observed [56,57]. These data sets would not be consistent with the approach adopted here. However, an explanation for the discrepancies observed among different experiments has not yet been found and therefore the debate on this point is still open.

### γH2AX signal kinetics and dose dependence: experimental and modelling aspects

In Fig 3 we showed that our modelling approach allows a consistent description of the **γ**H2AX signal kinetics as measured with flow cytometry. Specifically, after the normalization has been performed, clear dose dependence can be observed in the fit curves, which according to our approach is entirely due to DSB clustering at the micrometre scale. The quite extreme dose values employed are justified by the need to obtain a significant difference in the fraction of induced cDSB in order to be sensitive to the gradually delayed kinetics. In fact, cDSB represent only about 10% of the induced damage at 25 Gy, while the fraction increases up to about 95% at 500 Gy. Importantly, the best fit results are obtained when iDSB and cDSB are calculated assuming the standard domain size of 2 Mbp and including the 3D spreading mechanism. On the contrary, as shown in S4 Fig, the model fails to reproduce the kinetics at intermediate doses when only a larger domain size (8 Mbp) is considered. This suggests that the fractions of iDSB and cDSB resulting from a very large domain size do not allow an appropriate description of the kinetics data. This result is thus indirectly supportive for the existence of the 3D spreading mechanisms.

The concept of the enhanced severity of cDSB compared to iDSB has been discussed in more detail also in the recent paper by Hufnagl et al. [58]. Although the experimental approach adopted here does not allow drawing stronger conclusions, the indications obtained are of particular interest when combined to other recent analyses performed in our group [21,30]. Experimental evidence has been reported in the past for the relevance of micrometre size structures in the description of the induced DNA damage. For example, the studies performed by Johnston et al., where a delayed rejoining was attributed to the presence of multiple lesions in the same nuclear substructures [18,19]. At the same time, the analysis of fragment size distribution after irradiation reported by different groups, revealed an excess of fragments in the 1–2 Mbp size region which could be associated to the underlying presence of a loop organization [59,60]. In the same direction go the results obtained by Neumaier et al. [55] and by Vadhavkar et al [35]. DNA DSB complexity at different levels and the implications on lesion repair were considered in the recent review by Schipler and Iliakis [61]. Remarkably, they conclude that only the simultaneous presence of multiple DSB in close vicinity is likely to represent a more difficult task for the repair machinery, while no big differences are expected for simple DSB compared to DSB of enhanced chemical complexity or DSB originating from clustered non-DSB lesions.

A homogeneous distribution of DNA inside the nucleus is assumed in the GLOBLE. However, in experiments performed with low doses of low LET radiation, the fraction of DSB which is slowly repaired has been associated to the damage induced in the heterochromatic regions of the cell nucleus [62]. In such cases, the slow component of repair can be mainly associated to processing of the lesions by specific repair pathways (i.e. Homologous Recombination vs Non-Homologous End Joining). Although this approach has its merits, it has been recently shown that chromatin condensation alone is not sufficient to explain the differences observed in the repair kinetics after high LET irradiation, when therefore damage clustering and/or complexity are expected to play a major role [63]. This is also related to the results presented here. In fact, based only on the difference between eu- and heterochromatin, no difference would be expected in the repair kinetics when applying increasing doses of low LET radiation, in contrast with what observed in our data.

### γH2AX dose response and saturation: experimental and modelling aspects

The **γ**H2AX dose response curve as measured with flow cytometry was investigated in the past by MacPhail et al [9] up to a dose of 100 Gy. Different cell lines were considered, and for some of them the onset of saturation at high doses was observed. However, since the analysis of the saturation tendency was not the first aim of the paper, this aspect was not deeply discussed, neither complemented by a modelling analysis as it is here the case. Flow cytometry data of **γ**H2AX also appear in publications with different aims, but they are usually restricted to low doses (up to few Gy) and therefore not comparable with what we want to discuss here [64–66].

With respect to the saturation observed in the **γ**H2AX dose response curve, the results of our modelling analysis suggest that a good agreement to experimental data can be obtained following two different approaches. For instance, Fig 7 shows that a domain size larger than the standard 2 Mbp allows a reasonable description of the experimental data. At the same time, good agreement can be obtained starting with the 2 Mbp domain size and then simulating the 3D extension of the phosphorylation. However, the assumption of a very large domain size would strongly affect the fractions of induced iDSB and cDSB and thus the calculation of survival probabilities, while from this point of view good results have been obtained in the past by the GLOBLE assuming the standard size. Moreover, a domain size as large as 8–10 Mbp would not be supported by the current literature data. On the contrary, the 3D extension is not expected to affect lethality, being rather the result of signalling mechanisms. Thus, under this assumption the GLOBLE would be able to consistently reproduce both survival data as well as **γ**H2AX data as shown here.

**Fig 9:**
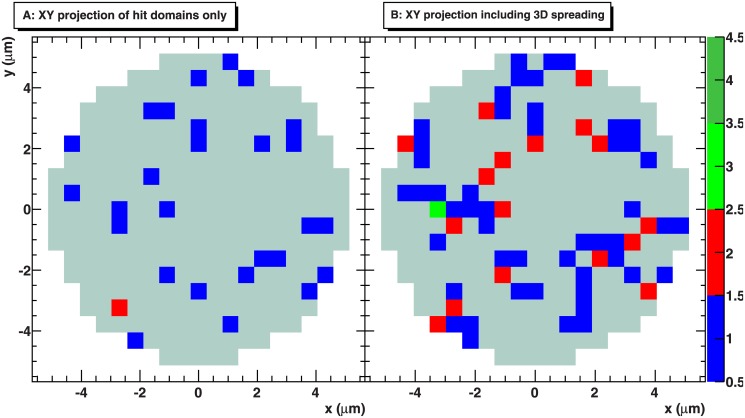
Predicted two-dimensional H2AX phosphorylation patterns with and without the 3D spreading mechanism. Example of XY projections of hit domains only (A) and of all phosphorylated domains obtained with the random expansion algorithm (B); the exposure to 1 Gy of X-rays photon radiation was simulated. The cell nucleus is represented in light grey in the background. The colour scale indicates the multiplicity of hit domains in the column of interest.

Concerning the relevance of the 3D spreading mechanisms, we extracted from the model calculations the Z projections of hit domains only and of all phosphorylated domains, as obtained when the random expansion algorithm is considered. The comparison between the two projections indicates that the overlap of neighbouring foci could affect the scoring of single foci. An example picture where the exposure to 1 Gy of photon radiation was simulated is shown in Fig 9. In panel A we can observe how the hit domains are distributed. This allows realizing that already at low doses it is possible that projected hit domains are close to each other, and that overlapping can take place in the xy plane as well as in z direction. By measuring the xy projection at the microscope, this second aspect would automatically translate in losing some DSB when scoring the foci. However, the problem can be partially overcome by using appropriate algorithms for the analysis which also increase the sensitivity in longitudinal direction. At the same time, in panel B we can observe how the fluorescence pattern would be in a manner confused by the 3D spreading of the H2AX phosphorylation, resulting in not well defined boundaries between single foci, in a distribution of foci sizes and in different local intensities.

A deeper investigation of the potential confounding factors in microscopy measurements related to the mechanisms of H2AX phosphorylation is beyond the scope of this work. However, similar conclusions indicating a loss of accuracy in the foci counting when the delivered dose exceeds few Gy, can be found in published works as well [7,8]. This consideration also fits to the saturation in the foci scoring which is usually observed already after few Gy of photon radiation [7,8,67].

All these aspects pooled together could finally contribute to determine an underscoring of the actual number of induced DSB, even at low doses which are of interest for instance in radiation protection. This observation is in line with the results of the modelling work presented by Ponomarev et al. and mentioned in the Introduction [34]. In their study a different approach is adopted, and the problem is tackled from the opposite direction, meaning that an algorithm is implemented which is able to estimate the actual number of DSB underlying the observed fluorescence pattern. This is based on the use of corrective factors which are estimated depending on the radiation quality, and it takes into account both the probability of having multiple DSB in the same focus, as well as the overlapping of close-by foci. Being the study limited to the consideration of doses up to few Gy, their approach is found to be relevant especially after high LET irradiation. However, it is reasonable to expect those mechanisms playing a substantial role also after high doses of low LET radiation.

### γH2AX dose response and saturation: the role of the DSB induction yield

In contrast to the choice of the domain size, the DSB induction yield is expected to be less dependent on the cell line under consideration [1]. Our results, suggesting an increased value for the DSB induction rate, fit to the recent work published by Neumaier et al [55]. In that study, live cell imaging of 53BP1 foci at low doses (0.1 Gy) was combined with a cumulative counting process, resulting in the determination of a DSB yield of 60–80 DSB/Gy. Even though a marker other than **γ**H2AX was employed, this approach is expected to partially solve the problems due to the kinetics of protein recruitment at DSB sites and to proximity effects (e.g. overlap of close-by foci). Moreover, the authors show that the DSB yield drops to about 30 DSB/Gy at 1 Gy, as the result of clustering of DSB into repair centres. This is actually in line with our conclusions. Nevertheless, these results contrast in a way with the value of 20–40 DSB/Gy, mainly resulting from gel elution experiments, which has been considered for many years as well established in the community [41,49]. However, some experimental issues affect gel electrophoresis techniques (e.g. only fragments having a length comprised between few kbp and few Mbp can be measured, and there is the need to use high doses, on the order of tens of Gy, to achieve reasonable sensitivity). Therefore, the debate is still open and the combination of modern experimental techniques with modelling studies could help to further elucidate this basic aspect of DNA damage induction, as indicated by the analysis presented here.

## Conclusions

With our model based on the GLOBLE approach, we were able to consistently describe the delayed disappearing of the **γ**H2AX signal for increasing doses, based on DSB clustering at the micrometre scale in the context of higher-order chromatin structures. These results, combined with recent works published by our group, support the relevance of the micrometre scale for the description and the interpretation of radiation induced biological effects. Furthermore, the outcome of our analysis on **γ**H2AX dose response curves is of interest concerning the use and the interpretation of **γ**H2AX data. In fact, when discussing the strong saturation observed in the measured signal, we showed that controversial interpretation of **γ**H2AX data may arise not only as a consequence of the frequently discussed technical issues, but also due to an incomplete understanding of the phosphorylation process. This observation might even extend to other markers involved in the DNA damage and repair processes. We found out that a substantial role might be attributed to 3D mechanisms of H2AX phosphorylation. More direct experimental support would be needed to confirm the importance of this kind of processes.

## Supporting Information

S1 Fig Comparison of dose response curve as measured with confocal microscopy pictures, and with flow cytometry AGD cells. Panel A: microscopy pictures showing the γH2AX staining pattern as observed at the confocal microscope for different doses 1 h after irradiation of AGD cells with X-rays (γH2AX in green and DNA in red are stained with Alexa488 and Topro-3, respectively); the “sum slices” Z-projection obtained with the ImageJ software is shown. Larger dark areas in the DNA staining which are free of γH2AX fluorescence represent nucleoli [68]. Panel B: the plot shows a direct comparison of the microscopy and flow cytometry signals normalized to the intensity measured at 50 Gy. Errors bars represent the standard error of the mean.
(EPS)

S2 Fig Modelling the γH2AX dose response: sensitivity analysis. The expected number of hit domains is calculated by assuming a fixed DSB induction rate equal to 30 DSB/Gy and by changing the domain size in a range from 2 to 10 Mbp (A); calculations are also shown, where the expected number of hit domains is simulated by assuming a fixed number of domains (3000 domains, corresponding to 2 Mbp/domain) and by changing the DSB induction rate inside a range comprised between 15 and 75 DSB/Gy (B).
(EPS)

S3 Fig Modellling analysis of γH2AX signal kinetics based on Poisson distribution and standard domain size of 2 Mbp. Experimental data are the same as shown in Fig 9. Lines show the result of the fit when input iDSB and cDSB are calculated according to Poisson distribution and assuming the standard domain size of 2 Mbp. Direct application to the experimental data (A) and comparison after normalization of the fit curves by maximum value for each dose are shown (B).
(EPS)

S4 Fig Modellling analysis of γH2AX signal kinetics based on Poisson distribution and enlarged domain size of 8 Mbp. Experimental data are the same as shown in Fig 9. Lines show the result of the fit when input iDSB and cDSB are calculated according to Poisson distribution and assuming an enlarged domain size of 8 Mbp. Direct application to the experimental data (A) and comparison after normalization of the fit curves by maximum value for each dose are shown (B). The curves relative to 250 and 500 Gy overlap in panel B, and this is the consequence of the cDSB fraction being close to 100% in both cases.
(EPS)

S1 Table γH2AX signal kinetics: fit parameters for standard domain size (2 Mbp) and Poisson distribution. Fit parameters resulting from the application of the γH2AX kinetic model to the experimental data shown in S3 Fig.
(DOC)

S2 Table γH2AX signal kinetics: fit parameters for enlarged domain size (8 Mbp) and Poisson distribution. Fit parameters resulting from the application of the γH2AX kinetic model to the experimental data shown in S4 Fig.
(DOC)
