# Supplementary material for: Induction and Processing of the Radiation-Induced Gamma-H2AX Signal and Its Link to the Underlying Pattern of DSB: A Combined Experimental and Modelling Study
Source: PLoS One. 2015 Jun 11;10(6):e0129416. doi: 10.1371/journal.pone.0129416 (PMC4465900; doi:10.1371/journal.pone.0129416)
Supplement: S2 Table — Fit parameters resulting from the application of the γH2AX kinetic model to the experimental data shown in S4 Fig. (DOC) [file pone.0129416.s006.doc]

| **D (Gy)** | **x_fluor_** |
| --- | --- |
| 2 | 0.048 ± 0.006 |
| 8 | 0.054 ± 0.004 |
| 25 | 0.048 ± 0.003 |
| 50 | 0.051 ± 0.002 |
| 250 | 0.081 ± 0.0002 |
| 500 | 0.088 ± 0.001 |
| τ | 0.22 ± 0.04 h |
| τ_fast_ | 1.37 ± 0.12 h |
| τ_slow_ | 6.51 ± 0.30 h |

Table S2
